# Multidrug-Resistant *Staphylococcus haemolyticus* ST42 Carrying ΨSCC*mec*_57395_-like SCC*mec* and Resistant Islands with Type I *aj1*–LP–*fusB* Structure Emerges in Taiwan Hospitals

**DOI:** 10.3390/antibiotics14101015

**Published:** 2025-10-13

**Authors:** Cheng-Mao Ho, Lee-Chung Lin, Yu-Hsiang Ou, Kai-Hsiang Lin, Jang-Jih Lu

**Affiliations:** 1 Division of Clinical Pathology, Taichung Tzu Chi Hospital, Buddhist Tzu Chi Medical Foundation, Taichung 427213, Taiwan; chengmao.ho@tzuchi.com.tw; 2 Division of Laboratory Medicine, Taichung Tzu Chi Hospital, Buddhist Tzu Chi Medical Foundation, Taichung 427213, Taiwan; tc102230@tzuchi.com.tw; 3 Department of Laboratory Medicine and Biotechnology, College of Medicine, Tzu Chi University, Hualien 970374, Taiwan; 4 Department of Pathology, College of Medicine, Tzu Chi University, Hualien 970374, Taiwan; 5 Department of Laboratory Medicine, Linkou Chang Gung Memorial Hospital, Taoyuan 333423, Taiwan; leollc@gmail.com (L.-C.L.); planet7668@cgmh.org.tw (Y.-H.O.); 6 Department of Life Sciences, National Chung Hsing University, Taichung 40227, Taiwan; 7 Division of Clinical Pathology, Taipei Tzu Chi Hospital, Buddhist Tzu Chi Medical Foundation, New Taipei 231405, Taiwan

**Keywords:** *S. haemolyticus*, *fusB*, *fusC*, ΨSCC*mec*_57395_, heavy metal resistance, mobile genetic elements

## Abstract

**Background/Objectives:** *Staphylococcus haemolyticus* is a common commensal bacterium that has emerged as an important nosocomial pathogen. Its multi-antibiotics resistance presents substantial therapeutic challenges in healthcare settings worldwide. Despite its growing clinical relevance, most investigations into antimicrobial resistance determinants have been focused on *Staphylococcus aureus* or *Staphylococcus epidermidis*, leaving *S. haemolyticus* comparatively understudied. This study aimed to elucidate the genetic basis of multi-drug resistance by characterizing mobile genetic elements associated with predominant *S. haemolyticus* clones circulating in Taiwan. **Methods:** From 2010 to 2017, 140 clinical targeted isolates of *S. haemolyticus* were obtained from individual patients. Two representative strains, SH53 (ST3) and SH51 (ST42), were sequenced using the PacBio^TM^ platform. The structural organization of SCC*mec* cassettes and phage-associated resistance islands in the remaining 138 isolates was analyzed by polymerase chain reaction (PCR) using specifically designed primers. **Results:** Of the 140 isolates, 92 (65.7%) were ST42 and 48 (34.3%) were ST3. PCR analysis showed that over two-thirds harbored heavy metal resistance genes. *cadD*, *cadX*, *arsC*, *arsB*, and *arsR* occurred in 90.2% of ST42 isolates, with *copA* in 71.7%. In ST3, these five genes were present in 89.6%, and *copA* in 64.6%. Fusidic acid (FA) resistance was more frequent in ST42 (46.7%) than ST3 (22.9%) (*p* = 0.015). Only one ST42 isolate carried *fusC*. The remaining 52 FA-resistant isolates contained a type I *aj1*–leader peptide (LP)–*fusB* structure downstream of *smpB*, except for a single ST42 isolate with the type IV structure. **Conclusions:** MDR ST42 *S. haemolyticus* carrying SCC*mec* cassettes with heavy metal resistance genes and phage-related islands carrying type I *aj1*–leader peptide (LP)–*fusB* structures may represent emerging opportunistic pathogens in Taiwan. Continued longitudinal surveillance is warranted to track the evolution of resistance-associated mobile elements under selective antimicrobial pressure.

## 1. Introduction

*Staphylococcus haemolyticus*, first identified in 1975, is a non-motile, non-spore-forming, catalase-positive, coagulase-negative, and Gram-positive coccus [1,2]. Similarly to *S. epidermidis*, *S. haemolyticus* is one of the most commensal normal floras in both humans and animals. Like other coagulase-negative staphylococci (CoNS), it is typically regarded as less virulent in healthy individuals due to its lack of coagulase, an enzyme that facilitates fibrin clot formation from fibrinogen and hinders host defense mechanisms [2,3]. This zoonotic microorganism can also be found in food and environmental sources [3]. Although often considered as harmless commensals, accumulating evidence indicates that *S. haemolyticus* contributes substantially to foreign-body or medical device–related infections, including bloodstream infections, arthritis, and valvular endocarditis in hospitalized and immunocompromised patients, including neutropenic patients, preterm neonates, and burn center patients with compromised skin or mucosal barriers [2,3,4].

In our previous reports, ST42 and ST3 were identified as the predominant multilocus sequence typing (MLST) lineages among clinically isolated *S. haemolyticus* and were associated with an outbreak of bacteremia in 36 patients at a burn center in Taiwan [4,5,6]. Of particular concern is their multidrug-resistant (MDR) profile, characterized by resistance to oxacillin, erythromycin, clindamycin, tetracycline, and fusidic acid (FA), which poses substantial therapeutic challenges, particularly for ST42 isolates [5,6].

The genetic determinants underlying MDR are frequently located on mobile genetic elements (MGEs), which facilitate horizontal transfer between bacteria via transduction, transformation, and conjugation/mobilization, including *S. haemolyticus* [7,8]. The MGEs include plasmids, *staphylococcal cassette chromosome mec* (SCC*mec*) cassettes, and phage-related resistance islands [2,3]. The intracellular and intercellular mobility of MGEs harboring resistance genes contributes to the widespread dissemination of multidrug-resistant bacteria, particularly under sustained antibiotic selective pressure [7]. Intracellular movement is mediated by insertion sequences, transposons, and integrons, whereas intercellular transfer involves mechanisms such as transformation, transduction, and conjugation/mobilization [7]. Among these *MGEs*, *SCCmec* is notable for conferring methicillin resistance in *Staphylococcus aureus* and coagulase-negative staphylococci. Two new SCC*mec* types, XIV and XV, were described recently [9,10,11].

In the era of extensive antimicrobial usage, MGEs carrying resistance genes are pivotal in driving the evolution and dissemination of MDR pathogens [7]. Under antibiotic selective pressure, MDR *S. haemolyticus* has emerged globally in hospital settings, especially ST42 strains in recent years [4,6,12,13,14]. However, the MGEs present in these MDR *S. haemolyticus* strains remain under-investigated. In this study, we aimed to characterize the MGEs carried by MDR *S. haemolyticus* isolates in Taiwan.

## 2. Results

### 2.1. MLST and Antimicrobial Resistance

Among the 140 *S. haemolyticus* isolates analyzed, MLST revealed that 92 strains belonged to ST42 and 48 strains to ST3. All isolates were resistant to penicillin, oxacillin, and erythromycin, and all remained susceptible to vancomycin (Table 1). Chloramphenicol resistance was more frequent in ST3 than in ST42 (14.6% vs. 4.3%, *p* = 0.048), whereas ST42 exhibited significantly higher resistance to tetracycline (70.7% vs. 4.3%, *p* < 0.0001) and fusidic acid (46.7% vs. 22.9%, *p* = 0.015).

### 2.2. The SCCmec Cassette Structures

**Figure 1:**
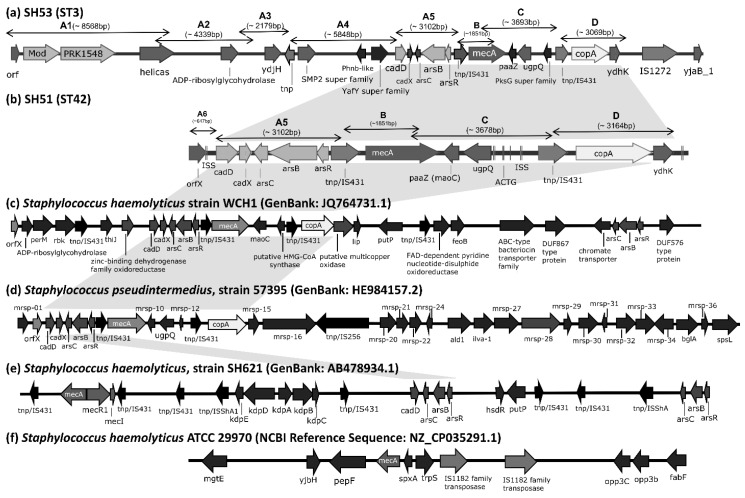
Structural organization of SCC*mec* cassettes in *S. haemolyticus* strains SH51, SH53, and other reference genomes, except *S. haemolyticus* strain ATCC 29970, which lacks a SCC*mec* cassette and genomic region surrounding the *mecA* locus is presented. Genome diagrams are not scaled proportionally to their actual sizes. Gray shading highlights structurally similar regions of heavy metal resistance genes shared across different strains. The pairwise sequence similarities among SCC*mec* cassettes from SH51, SH53, and reference genomes are provided in the Supplementary Material Table S1-1. mrsp-01: metallo-beta-lactamase superfamily proteins; mrsp-16: putative type I DNA modification methylase; mrsp-27: amino acid permease; mrsp-28: lead, cadmium, zinc and mercury transporting ATPase; mrsp-29: transcriptional regulator; mrsp-30: putative type II restriction endonuclease, DpnII superfamily; mrsp-32: putative restriction modification DNA methylase; mrsp-33: putative DNA methylase; mrsp-34: GntR family transcriptional regulator; mrsp-10, 12, 15, 31, 36: hypothetical protein; cadD: cadmium binding protein; cadX: cadmium resistant accessory protein; arsC: arsenate reductase; arsB: arsenical pump membrane protein; arsR: arsenical resistance operon repressor; copA: copper-translocating P-type ATPase.

Whole-genome sequencing showed that the ST42 isolate SH51 harbored a SCC*mec* cassette approximately 12,000 bp in size, comprising *orfX*, heavy-metal resistance genes (*cadD*, *cadX*, *arsC*, *arsB*, *arsR*), *IS431*, *mecA*, *paaZ*, *ugpQ*, *IS431*, *copA*, and *ydhK* (Figure 1b). In contrast, the ST3 isolate SH53 possessed a larger SCC*mec* cassette (~32,000 bp), including *orfX*, *Mod*, *PRK15483*, *helicase*, *ADP-ribosylglycohydrolase*, *ydjH*, SMP2 superfamily, YafY superfamily, heavy-metal resistance genes (*cadD*, *cadX*, *arsC*, *arsB*, *arsR*), *IS431*, *mecA*, *paaZ*, *ugpQ*, PksG superfamily, *IS431*, *copA*, and *ydhK* (Figure 1a). In GenBank, the SCC*mec* cassette of *S. haemolyticus* strain WCH1 (GenBank: JQ764731.1) and *Staphylococcus pseudintermedius* strain 57395 (GenBank: HE984157.2) encode all of the aforementioned heavy-metal resistance genes (*cadD*, *cadX*, *arsC*, *arsB*, *arsR*, and *copA*) (Figure 1c and Figure 1d, respectively), whereas *S. haemolyticus* strain SH621 (GenBank: AB478934.1) contains only *cadD*, *arsC*, *arsB*, and *arsR*, lacking *cadX* and *copA* (Figure 1e). Notably, *S. haemolyticus* strain ATCC29970 (RefSeq: NZ_CP035291.1) carries *mecA* only and lacks other SCCmec-associated components (Figure 1f).

**Figure 2:**
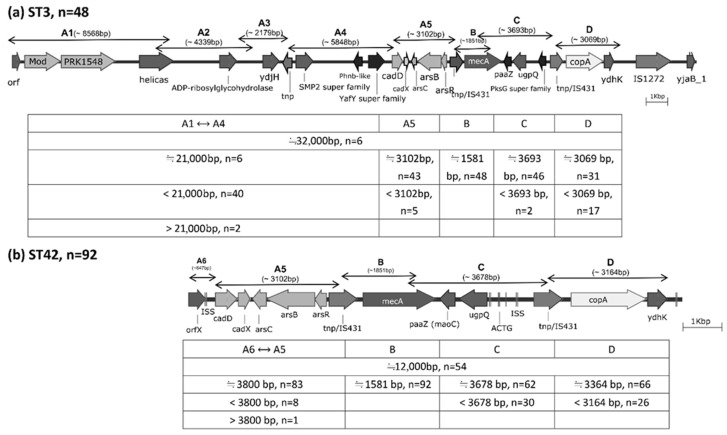
Distribution of gene fragments amplified by nine primer pairs, designed based on the SCC*mec* cassettes of *S. haemolyticus* strains SH53 (ST3) and SH51 (ST42), as determined by whole-genome sequencing.

Based on PCR product sizes obtained using five primer pairs (A5, A6, B, C, and D), 53 ST42 strains (53/92, 57.6%) exhibited SCC*mec* cassette structures similar to that of strain SH51. With the exception of *mecA*, the remaining ST42 isolates carried heterogeneous gene fragments of varying lengths (38/92, 41.3%; see Figure 2b). Notably, over two-thirds of these isolates contained full-length heavy metal resistance genes comparable to those in SH51: *cadD*, *cadX*, *arsC*, *arsB*, and *arsR* were detected in 83/92 strains (90.2%), while 66/92 strains (71.7%) carried *copA*. Among the 47 ST3 strains analyzed, five (5/48, 10.4%) possessed SCC*mec* structures resembling that of strain SH53 (Figure 2a). Aside from *mecA* and two strains containing larger gene structures spanning regions A1 to A4, the remaining 40 strains exhibited truncated or reduced variants of the SCC*mec* cassette components. As observed in ST42, the majority of ST3 strains also carried full-length heavy metal resistance genes: 43/48 (89.6%) harbored *cadD*, *cadX*, *arsC*, *arsB*, and *arsR*, whereas 31/48 (64.6%) possessed *copA*. Overall, there were no significant differences between ST3 and ST42 isolates in the carriage of heavy metal resistance gene segments, despite their high prevalence (Table 1). Detailed SCC*mec* cassette comparisons among *S. haemolyticus* strains SH51, SH53, and selected reference genomes are provided in the Supplementary Material Table S1-1.

### 2.3. Phage-Related FA Resistance Islands

**Figure 3:**
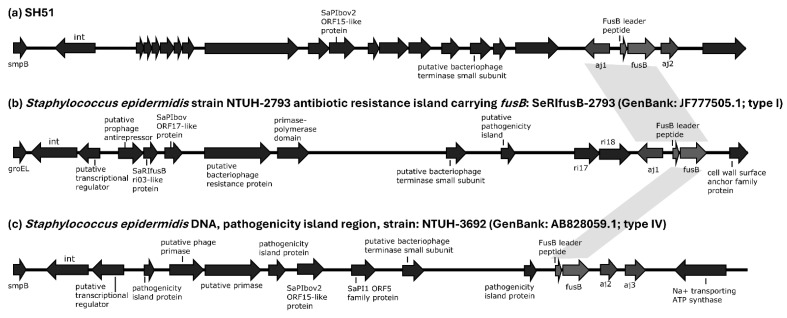
Structural organization of phage-related fusidic acid resistance islands in *S. haemolyticus* strain SH51 and selected reference genomes. Genome diagrams are not proportionally scaled to actual sequence lengths. Gray shading denotes type 1 and type 4 *aj1*–leader peptide (LP)–*fusB* configurations shared across SH51 and reference strains. The sequence similarity of phage-related fusidic acid resistance islands among SH51, and other reference strains is detailed in the Supplementary Material Table S1-2.

FA resistance was also more prevalent in ST42 isolates (43/92, 46.7%) than in ST3 isolates (11/48, 22.9%). Among these, only one ST42 isolate carried *fusC*, and one ST3 strain harbored a type IV *fusB* structure. The remaining 52 FA-resistant isolates all carried a type I *aj1*–leader peptide (LP)*–fusB* structure inserted downstream of *smpB*, rather than *groEL* or *rpsR* (Figure 3a). Detailed comparative analyses of phage-related fusidic acid resistance islands between SH51 and other reference genomes are provided in the Supplementary Material Figure S1.

## 3. Discussion

In our previous reports, ST42 (59.8%) and ST3 (20.6%) were the predominant MLST lineages among clinical *S. haemolyticus* isolates, particularly those from burn center patients [5]. The two sequence types differ at the SH1431 locus among the seven housekeeping genes used in MLST, and phylogenetic analysis of MLST allelic profiles indicated a close relationship between ST42 and ST3 [5]. In the present study, an even greater proportion of ST42 isolates (65.7%) was observed, and the ST42 lineage has recently been reported as an emerging nosocomial pathogen in China [14]. Moreover, the antimicrobial resistance rates among these 140 *S. haemolyticus* isolates, against penicillin, oxacillin, erythromycin, clindamycin, and trimethoprim–sulfamethoxazole, exceeded those reported previously [12,13]. The ST42 lineage, in particular, exhibited significantly higher resistance to tetracycline, fusidic acid, and trimethoprim–sulfamethoxazole. This MDR phenotype may facilitate dissemination in the era of widespread antibiotic use, although vancomycin remains a clinically useful therapeutic option.

Among these 140 isolates, more than 60% harbored heavy metal resistance genes, including *cadD*, *cadX*, *arsC*, *arsB*, *arsR*, and *copA*. The determinants for copper (*copA*), arse-nic (*arsC*, *arsB*, *arsR*), and cadmium (*cadD*, *cadX*) were associated with elevated minimum inhibitory concentrations (MICs) for the respective metals [15]. *CopA*, encoding a copper-translocating P-type ATPase, is suspected to mediate efflux and maintain intracellular copper homeostasis in staphylococci [15]. The functions of the arsenic resistance determinants are as follows: *arsC* encodes an arsenate reductase that reduces intracellular As^5+^ to As^3+^; *arsB* encodes an arsenical pump membrane protein responsible for exporting As^3+^ from the cytosol to the periplasmic or extracellular space; and *arsR* encodes an operon repressor that regulates transcription of the *ars* operon [16,17]. Regarding cadmium resistance, *cadD* encodes an energy-dependent efflux pump (cadmium-binding protein) that contributes to increased Cd^2+^ MICs, while *cadX*, a cadmium-resistance accessory protein, regulates *cadD* expression by binding to its promoter region [17,18]. Several staphylococcal species, including *S. aureus*, *S. haemolyticus*, *S. saprophyticus*, *S. epidermidis*, *S. warneri*, and *S. pseudintermedius*, were reported to carry these heavy metal resistance genes [10,15,17,19,20,21]. Notably, the SCC*mec* structures of *S. haemolyticus* strains SH51 and SH53 closely resemble the pseudo-Staphylococcal Cassette Chromosome *mec* element (ΨSCC*mec*_57395_) from *S. pseudintermedius* strain 57395 in Thailand (GenBank: HE984157.2, Figure 1c) and *S. haemolyticus* strain WCH1 in China (GenBank: JQ764731.1) [17,21]. This study is the first report of ΨSCC*mec*_57395_ in clinically significant *S. haemolyticus* isolates from Taiwan. These findings suggest that this MGE has disseminated across diverse *Staphylococcus* species and geographic regions, underscoring its potential role in the global transmission of methicillin resistance. Heavy metal resistance genes frequently coexist with antibiotic resistance determinants on the same mobile genetic elements (e.g., SCC*mec*), which may promote the evolution and dissemination of DMR bacterial strains [15,22].

Currently, determinants associated with fusidic acid resistance include *fusA*, *fusB*, *fusC*, *fusD*, *fusE*, and *fusF* [23,24]. Mutations in *fusA*, which encodes the drug target elongation factor G (EF-G), can lead to high-level resistance (e.g., MIC > 64 μg/mL). In contrast, the small protein products encoded by *fusB*, *fusC*, *fusD*, and *fusF* bind to EF-G and interfere with fusidic acid binding, resulting in low-level resistance [23,24]. Additionally, mutation of *rplF*, encoding ribosomal protein L6, a contact site for EF-G, leads to *fusE*, which is associated with small-colony variants of *S. aureus* exhibiting low-level fusidic acid resistance [25]. In this study, *fusB* accounted for the majority of fusidic acid resistance among *S. haemolyticus* isolates (53/54, 98.1%), with the remaining one isolate carrying *fusC*. Horizontal gene transfer of *fusB* among *Staphylococcus* species can occur via plasmids, transposon-like elements, or pathogenicity islands, contributing to clonal dissemination [26]. Previous reports showed that resistance islands carrying *fusB* frequently integrate at *smpB*, *groEL*, or *rpsR* loci in *S. epidermidis* isolates. Four structural types of the *aj1*–leader peptide (LP)*–fusB* region were described (Figure 3 and Figure S1): full-length *aj1* (type I), partial *aj1* fragments (type II), truncated *aj1* (type III), and structures lacking *aj1* (type IV) [27,28,29]. Among the 53 *fusB*-positive *S. haemolyticus* isolates in this study, all carried type I *aj1*–LP–*fusB* structures inserted downstream of *smpB*; the exception was a single ST3 strain that possessed a type IV variant. The predominance of type I *aj1*–LP–*fusB* structures within resistance islands harboring *fusB* in clinical *S. haemolyticus* isolates has not been previously reported.

This study has several limitations. The isolates were collected from only two hospitals, which may limit the generalizability of the findings. Whole-genome sequencing was performed for only two representative strains (SH51 and SH53), which would reduce the reliability of genomic comparisons. The uneven enrollment of ST42 (*n* = 92) and ST3 (*n* = 48) strains may introduce bias in results interpretation. Detailed clinical information, such as underlying diseases, antimicrobial therapies, and patient outcomes, was not available, limiting the study’s clinical applicability. Future work should include multi-hospital sampling, comprehensive clinical characteristics and outcomes surveillance, and broader investigation of mobile genetic elements in both clinically significant and commensal *S. haemolyticus* and other coagulase-negative staphylococci. Despite these constraints, the data indicate that the predominant *S. haemolyticus* clones circulating in nosocomial environments in Taiwan are ST3 and ST42. Most strains carried ΨSCC*mec*_57395_, which harbors heavy metal resistance genes, including *cadD*, *cadX*, *arsC*, *arsB*, *arsR*, and *copA*, similar to those described in *S. pseudintermedius* strain 57395 and *S. haemolyticus* strain WCH1. The coexistence of heavy metal resistance genes and antibiotic resistance genes within the same mobile genetic elements may contribute to enhanced adaptability of these strains in hospital environments with antimicrobial and heavy metal exposure [15,22]. Among these dominant lineages, resistance islands integrated downstream of *smpB*, carrying a type I *aj1–LP–fusB* structure, accounted for fusidic acid resistance in most isolates.

## 4. Materials and Methods

### 4.1. Enrolled Isolates, Antimicrobial Testing and MLST

Between 2010 and 2017, a total of 140 clinical isolates of *S. haemolyticus* were recovered from clinically indicated cultures of individual patients and identified using the Bruker MALDI Biotyper^®^ system (Bruker Daltonik GmbH, Bremen, Germany). Antimicrobial susceptibility testing were performed by the disc diffusion method on Mueller–Hinton agar (Nippon Becton Dickinson Company, Ltd., Tokyo, Japan) according to Clinical and Laboratory Standards Institute (CLSI) guidelines [30], except vancomycin which susceptibility was determined using the ETEST^®^ (bioMérieux, Marcy-l’Étoile, France). *Staphylococcus aureus* ATCC^®^ 25923 was used as the quality control strain, and the results interpretation followed CLSI recommendations [31], whereas the FA results were interpreted according to the European Committee on Antimicrobial Susceptibility Testing (EUCAST) criteria [32]. BD BBL™ Sensi-Disc™ antimicrobial susceptibility test discs (Becton Dickinson, Sparks, MD, USA) were used for the disc diffusion assays except vancomycin. These strains were collected from two hospitals in Taiwan, with 92 isolates from a northern hospital and 48 from a southern hospital. This study was approved by the Biosafety Committee of Chang Gung Medical Foundation (Approval No. 00417-2020092285432). MLST of each *S. haemolyticus* isolate was determined based on the sequences of seven housekeeping genes: *arc*, *SH1200*, *hemH*, *leuB*, *SH1431*, *cfxE*, and *RiboseABC*, as previously described [33]. The sequence types (STs) were assigned via the PubMLST database (https://pubmlst.org/shaemolyticus/) (accessed on 1 July 2022) [34].

### 4.2. MGEs Analysis

Genomic DNA from two representative *S. haemolyticus* strains—SH51 (ST42; accession numbers CP092478 and CP092479) and SH53 (ST3; accession numbers CP092476 and CP092477)—was extracted using the Puregene Yeast/Bact. Kit B (QIAGEN Sciences, Germantown, MD, USA). Whole-genome sequencing was performed on the PacBio™ sequencing platform (Pacific Biosciences, Menlo Park, CA, USA). Libraries were prepared using the standard SMRTbell protocol without PCR amplification (PCR-free); therefore, no technical PCR replicates were generated. Assemblies were polished and circularized, and the final Circlator builds were used for all analyses. Final assembly and quality control statistics were as follows: isolate SH51: 2 circular contigs; total length, 2,596,982 bp; largest contig, 2,563,044 bp; N50, 2,563,044 bp; GC content, 33%; isolate SH53: 2 circular contigs; total length, 2,586,626 bp; largest contig, 2,536,380 bp; N50, 2,536,380 bp; GC content, 33%. In both genomes, the N50 equaled the largest contig, consistent with a near-complete chromosome plus one smaller circular element. Antimicrobial resistance genes in these strains were identified from the sequencing data using basic local alignment search tool (BLAST)-based searches against the ResFinder database [35] and Comprehensive Antibiotic Resistance Database (CARD) [36]. Comparative analyses between these two strains and other reference genomes were performed using the blastn mode of the BLAST+ command-line suite (https://blast.ncbi.nlm.nih.gov/doc/blast-help/downloadblastdata.html#downloadblastdata, accessed on 1 July 2022).

#### 4.2.1. SCC*mec* Cassettes Analysis

Based on the whole-genome sequencing results of the two selected strains, nine primer pairs (A1 to A6, B, C, and D) were designed to investigate the structural organization of the SCC*mec* cassettes in the remaining 138 *S. haemolyticus* isolates (Table S2). Each PCR assay was performed at least twice, with SH51 or SH52 included as controls depending on their MLST type.

#### 4.2.2. Phage-Related Islands and FA Resistance Determinants Analysis

FA-resistant isolates were screened by PCR for the presence of *fusB* and *fusC* using previously described primers (Table 1) [26,29]. To investigate phage-associated resistance island structures among *fusB*-positive isolates, four primer pairs from earlier studies were employed to determine insertion sites (*smpB*, *groEL*, or *rpsR*) and to classify the *aj1*–leader peptide (LP)–*fusB* region into four structural types (Table 1) [27,28,29].

### 4.3. Statistical Analysis

Pearson’s chi-square test or Fisher’s exact test (when the expected frequency in any cell was <5) was employed to evaluate the statistical significance of differences between ST3 and ST42 isolates. All statistical analyses were conducted using the Statistical Package for the Social Sciences (SPSS) for Windows, version 17.0 (Chicago, IL, USA). A *p*-value of ≤0.05 was considered indicative of statistical significance.

## 5. Conclusions

With the growing number of elderly and debilitated patients in Taiwan, as reported in the Taiwan Health and Welfare Report 2023 and in our previous studies [4,6,37], MDR ST42 *S. haemolyticus* harboring ΨSCC*mec*_57395_-like SCC*mec* with heavy metal resistance genes and phage-related islands carrying type I *aj1*–leader peptide (LP)–*fusB* structures may represent emerging opportunistic pathogens in Taiwan. Continuous surveillance of mobile genetic elements carrying antibiotic resistance genes in clinical *Staphylococcus* isolates is essential to elucidate their evolutionary dynamics and multidrug resistance potential under the selective pressure of widespread antibiotic use.

## Figures and Tables

**Table 1:** Comparison of antimicrobial resistance and heavy metal resistance gene segments between ST3 and ST42 among 140 *S. haemolyticus* isolates.

Antimicrobial Agents	ST3 (*n* = 48)	ST42 (*n* = 92)	*p* Value ^a^
Penicillin	100%	100%	-
Oxacillin	100%	100%	-
Clindamycin	66.7%	60.9%	0.53
Erythromycin	100%	100%	-
Tetracycline	4.3%	70.7%	<0.0001 *
Chloramphenicol	14.6%	4.3%	0.048 *
Vancomycin	0%	0%	-
Fusidic acid	22.9%	46.7%	0.015 *
Trimethoprim-sulfamethoxazole	62.5%	76.1%	0.12
Heavy-metal-resistant gene segments with similar size to SH53 or SH51 strains	ST3 (*n* = 48)	ST42 (*n* = 92)	*p* value
A5/A5-A6 segment (*cadD*, *cadX*, *arsC*, *arsB*, *arsR*)	89.6%	90.2%	0.91
D segment (*copA*)	64.6%	71.7%	0.39

^a^ *p* value by Fisher’s exact test when the cell expectation was less than five. * *p* ≤ 0.05.

## Data Availability

The data supporting the findings of this study are contained within the article and its Supplementary Materials.

## Supplementary Materials

The following supporting information can be downloaded at: https://www.mdpi.com/article/10.3390/antibiotics14101015/s1, Figure S1: Structural organization of phage-related fusidic acid resistance islands in *S. haemolyticus* reference genomes; Table S1-1: Comparative analyses of SCC*mec* cassettes between SH51, SH53 and other reference genomes using the blastn mode of the BLAST+ command-line suite; Table S1-2: Comparative analyses of phage-related fusidic acid resistance islands between SH51 and other reference genomes using the blastn mode of the BLAST+ command-line suite; Table S2: Primers used in this study.

## Abbreviations

The following abbreviations are used in this manuscript:

SCC*mec*	Staphylococcal cassette chromosome *mec*
MLST	multilocus sequence typing
MDR	multidrug-resistant
ST	sequence type
PCR	polymerase chain reaction
LP	leader peptide
CoNS	coagulase-negative staphylococci
MGEs	mobile genetic elements
BLAST	basic local alignment search tool
CARD	Comprehensive Antibiotic Resistance Database
FA	fusidic acid
