# Supplementary material for: Multidrug-Resistant Staphylococcus haemolyticus ST42 Carrying ΨSCCmec57395-like SCCmec and Resistant Islands with Type I aj1–LP–fusB Structure Emerges in Taiwan Hospitals
Source: Antibiotics (Basel). 2025 Oct 13;14(10):1015. doi: 10.3390/antibiotics14101015 (PMC12561750; doi:10.3390/antibiotics14101015)
Supplement: Supplementary file 1 [file antibiotics-14-01015-s001.zip › Figure S1 .pdf]

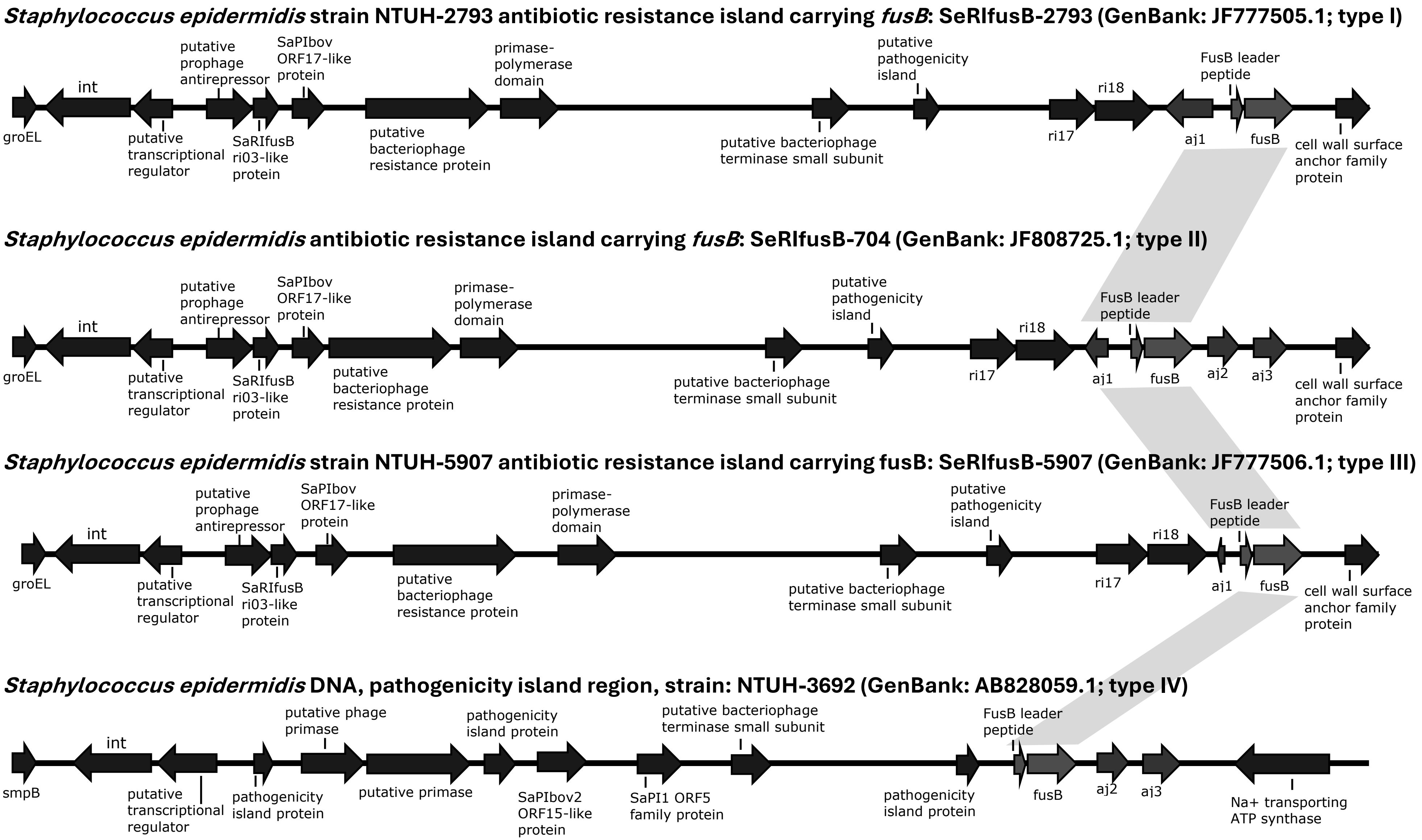

Figure S1. Structural organization of phage-related fusidic acid resistance islands in *S. haemolyticus* reference genomes. Genome diagrams are not proportionally scaled to actual sequence lengths. Gray shading denotes four distinct *aj1*–leader peptide (LP)–*fusB* configurations shared across different strains. The sequence similarity of phage-related fusidic acid resistance islands among SH51, and other reference strains is detailed in the Supplementary Table S1-2.
