# Supplementary material for: Multidrug-Resistant Staphylococcus haemolyticus ST42 Carrying ΨSCCmec57395-like SCCmec and Resistant Islands with Type I aj1–LP–fusB Structure Emerges in Taiwan Hospitals
Source: Antibiotics (Basel). 2025 Oct 13;14(10):1015. doi: 10.3390/antibiotics14101015 (PMC12561750; doi:10.3390/antibiotics14101015)
Supplement: Supplementary file 1 [file antibiotics-14-01015-s001.zip › Table S1.pdf]

Table S1-1 Comparative analyses of SCC*mec* cassettes betweenSH51, SH53 and other reference genomes using the blastn mode of the BLAST+ command-line suite.

query id	subject id	% identity	alignment length	mismatches	gap openings	q.start	q.end	s.start	s.end	e-value	bit score
SH51-SCC*mec*	SH53-SCC*mec*	99.719	9615	12	1	463	10062	20404	30018	0	17592
SH51-SCC*mec*	SH53-SCC*mec*	99.845	7094	2	1	10158	17251	30019	37103	0	13031
SH51-SCC*mec*	SH53-SCC*mec*	97.425	1437	37	0	21207	22643	20418	21854	0	2449
SH51-SCC*mec*	SH53-SCC*mec*	84.513	1808	266	13	22669	24469	21844	23644	0	1775
SH51-SCC*mec*	SH53-SCC*mec*	99.875	797	1	0	3775	4571	29228	30024	0	1467
SH51-SCC*mec*	SH53-SCC*mec*	100	791	0	0	9272	10062	23716	24506	0	1461
SH51-SCC*mec*	SH53-SCC*mec*	99.247	797	5	1	9273	10068	15412	14616	0	1437
SH51-SCC*mec*	SH53-SCC*mec*	99.494	790	4	0	3776	4565	15412	14623	0	1437
SH51-SCC*mec*	SH53-SCC*mec*	98.98	490	5	0	1	490	1	490	0	878
SH51-SCC*mec*	SH53-SCC*mec*	88.564	411	45	2	8253	8662	28154	28563	5.96E-141	497
SH51-SCC*mec*	SH53-SCC*mec*	88.321	411	46	2	8213	8622	28194	28603	2.77E-139	492
SH51-SCC*mec*	SH53-SCC*mec*	88.919	370	41	0	8293	8662	28154	28523	1.01E-128	457
SH51-SCC*mec*	SH53-SCC*mec*	88.919	370	41	0	8213	8582	28234	28603	1.01E-128	457
SH51-SCC*mec*	SH53-SCC*mec*	89.489	333	27	7	8334	8662	28155	28483	6.18E-116	414
SH51-SCC*mec*	SH53-SCC*mec*	89.189	333	28	7	8214	8542	28275	28603	2.87E-114	409
SH51-SCC*mec*	SH53-SCC*mec*	88.66	291	33	0	8372	8662	28153	28443	3.80E-98	355
SH51-SCC*mec*	SH53-SCC*mec*	88.66	291	33	0	8212	8502	28313	28603	3.80E-98	355
SH51-SCC*mec*	SH53-SCC*mec*	90.094	212	19	2	8212	8422	28393	28603	1.09E-73	274
SH51-SCC*mec*	SH53-SCC*mec*	88.304	171	16	3	8214	8382	28435	28603	5.24E-52	202
SH51-SCC*mec*	SH53-SCC*mec*	100	28	0	0	13095	13122	463	490	5.58E-07	52.8
SH51-SCC*mec*	SHWCH1-JQ764731.1	98.918	10446	96	3	1901	12330	9822	20266	0	18648
SH51-SCC*mec*	SHWCH1-JQ764731.1	95.856	7143	264	14	17350	24469	4491	11624	0	11522
SH51-SCC*mec*	SHWCH1-JQ764731.1	99.488	2344	12	0	22462	24805	38879	41222	0	4263
SH51-SCC*mec*	SHWCH1-JQ764731.1	97.427	1438	33	2	477	1913	8364	9798	0	2447
SH51-SCC*mec*	SHWCH1-JQ764731.1	84.475	1810	263	16	1903	3703	39086	40886	0	1770
SH51-SCC*mec*	SHWCH1-JQ764731.1	100	791	0	0	9272	10062	11696	12486	0	1461
SH51-SCC*mec*	SHWCH1-JQ764731.1	100	791	0	0	3775	4565	17208	17998	0	1461
SH51-SCC*mec*	SHWCH1-JQ764731.1	98.357	791	13	0	3775	4565	3700	4490	0	1389
SH51-SCC*mec*	SHWCH1-JQ764731.1	98.357	791	13	0	9272	10062	3700	4490	0	1389
SH51-SCC*mec*	SHWCH1-JQ764731.1	98.093	734	9	5	12404	13133	21727	22459	0	1273
SH51-SCC*mec*	SHWCH1-JQ764731.1	92.528	803	48	5	9273	10064	30690	29889	0	1140
SH51-SCC*mec*	SHWCH1-JQ764731.1	92.509	801	48	5	3776	4565	30690	29891	0	1136
SH51-SCC*mec*	SHWCH1-JQ764731.1	93.251	726	46	2	9342	10065	26462	27186	0	1066
SH51-SCC*mec*	SHWCH1-JQ764731.1	93.223	723	46	2	3845	4565	26462	27183	0	1061
SH51-SCC*mec*	SHWCH1-JQ764731.1	98.726	314	4	0	165	478	1	314	2.70E-159	558
SH51-SCC*mec*	SHWCH1-JQ764731.1	88.808	411	44	2	8253	8662	16134	16543	1.28E-142	503
SH51-SCC*mec*	SHWCH1-JQ764731.1	88.564	411	45	2	8213	8622	16174	16583	5.96E-141	497
SH51-SCC*mec*	SHWCH1-JQ764731.1	89.189	370	40	0	8293	8662	16134	16503	2.18E-130	462
SH51-SCC*mec*	SHWCH1-JQ764731.1	89.189	370	40	0	8213	8582	16214	16583	2.18E-130	462
SH51-SCC*mec*	SHWCH1-JQ764731.1	89.79	333	26	7	8334	8662	16135	16463	1.33E-117	420
SH51-SCC*mec*	SHWCH1-JQ764731.1	89.489	333	27	7	8214	8542	16255	16583	6.18E-116	414

SH51-SCC*mec*	SHWCH1-JQ764731.1	89.003	291	32	0	8212	8502	16293	16583	8.16E-100	361
SH51-SCC*mec*	SHWCH1-JQ764731.1	88.316	291	34	0	8372	8662	16133	16423	1.77E-96	350
SH51-SCC*mec*	SHWCH1-JQ764731.1	90.094	212	19	2	8212	8422	16373	16583	1.09E-73	274
SH51-SCC*mec*	SHWCH1-JQ764731.1	88.304	171	16	3	8214	8382	16415	16583	5.24E-52	202
SH51-SCC*mec*	SP57395-HE984157.2:1515-14258	98.544	8930	120	7	1	8925	1	8925	0	15762
SH51-SCC*mec*	SP57395-HE984157.2:1515-14258	99.922	3824	0	3	9271	13094	8924	12744	0	7042
SH51-SCC*mec*	SP57395-HE984157.2:1515-14258	97.286	1437	39	0	21207	22643	477	1913	0	2438
SH51-SCC*mec*	SP57395-HE984157.2:1515-14258	84.513	1808	266	13	22669	24469	1903	3703	0	1775
SH51-SCC*mec*	SP57395-HE984157.2:1515-14258	100	791	0	0	9272	10062	3775	4565	0	1461
SH51-SCC*mec*	SP57395-HE984157.2:1515-14258	99.625	801	2	1	3766	4565	8915	9715	0	1461
SH51-SCC*mec*	SP57395-HE984157.2:1515-14258	89.781	411	40	2	8253	8662	8213	8622	2.74E-149	525
SH51-SCC*mec*	SP57395-HE984157.2:1515-14258	89.051	411	43	2	8213	8622	8253	8662	2.76E-144	508
SH51-SCC*mec*	SP57395-HE984157.2:1515-14258	89.488	371	37	2	8213	8582	8293	8662	4.68E-132	468
SH51-SCC*mec*	SP57395-HE984157.2:1515-14258	88.679	371	40	2	8293	8662	8213	8582	4.71E-127	451
SH51-SCC*mec*	SP57395-HE984157.2:1515-14258	90.09	333	25	7	8214	8542	8334	8662	2.85E-119	425
SH51-SCC*mec*	SP57395-HE984157.2:1515-14258	89.79	333	26	7	8334	8662	8214	8542	1.33E-117	420
SH51-SCC*mec*	SP57395-HE984157.2:1515-14258	88.66	291	33	0	8372	8662	8212	8502	3.80E-98	355
SH51-SCC*mec*	SP57395-HE984157.2:1515-14258	88.66	291	33	0	8212	8502	8372	8662	3.80E-98	355
SH51-SCC*mec*	SP57395-HE984157.2:1515-14258	89.623	212	20	2	8212	8422	8452	8662	5.09E-72	268
SH51-SCC*mec*	SP57395-HE984157.2:1515-14258	87.719	171	17	3	8214	8382	8494	8662	2.44E-50	196
SH51-SCC*mec*	SH621-AB478934.1	95.889	7200	264	14	17293	24469	29647	36837	0	11627
SH51-SCC*mec*	SH621-AB478934.1	99.731	4096	11	0	4567	8662	7096	3001	0	7504
SH51-SCC*mec*	SH621-AB478934.1	99.512	3074	12	3	10063	13133	36886	39959	0	5590
SH51-SCC*mec*	SH621-AB478934.1	99.423	2252	10	3	22462	24710	55042	57293	0	4084
SH51-SCC*mec*	SH621-AB478934.1	96.697	1847	59	2	1901	3746	35035	36880	0	3072
SH51-SCC*mec*	SH621-AB478934.1	94.004	1868	79	7	8213	10062	3410	1558	0	2798
SH51-SCC*mec*	SH621-AB478934.1	97.566	1438	31	2	477	1913	33577	35011	0	2459
SH51-SCC*mec*	SH621-AB478934.1	84.577	1809	264	14	1903	3703	55249	57050	0	1781
SH51-SCC*mec*	SH621-AB478934.1	98.866	794	9	0	9273	10066	14908	14115	0	1417
SH51-SCC*mec*	SH621-AB478934.1	98.987	790	8	0	3776	4565	14908	14119	0	1415
SH51-SCC*mec*	SH621-AB478934.1	98.492	796	11	1	3776	4570	28856	29651	0	1402
SH51-SCC*mec*	SH621-AB478934.1	98.609	791	10	1	9273	10062	28856	29646	0	1399
SH51-SCC*mec*	SH621-AB478934.1	97.484	795	17	3	9270	10062	10194	9401	0	1354
SH51-SCC*mec*	SH621-AB478934.1	97.478	793	17	3	3775	4565	10192	9401	0	1351
SH51-SCC*mec*	SH621-AB478934.1	93.577	794	48	3	9273	10064	48180	47388	0	1181
SH51-SCC*mec*	SH621-AB478934.1	93.561	792	48	3	3776	4565	48180	47390	0	1177
SH51-SCC*mec*	SH621-AB478934.1	92.812	793	41	4	3775	4565	2336	1558	0	1134
SH51-SCC*mec*	SH621-AB478934.1	93.388	726	45	2	9342	10065	43962	44686	0	1072
SH51-SCC*mec*	SH621-AB478934.1	93.361	723	45	2	3845	4565	43962	44683	0	1066
SH51-SCC*mec*	SH621-AB478934.1	100	478	0	0	1	478	187	664	0	883
SH51-SCC*mec*	SH621-AB478934.1	90.732	410	38	0	8213	8622	3370	2961	5.84E-156	547
SH51-SCC*mec*	SH621-AB478934.1	89.294	411	42	2	8253	8662	3450	3041	5.92E-146	514
SH51-SCC*mec*	SH621-AB478934.1	89.488	371	35	4	8214	8582	3329	2961	1.68E-131	466
SH51-SCC*mec*	SH621-AB478934.1	89.189	370	40	0	8293	8662	3450	3081	2.18E-130	462
SH51-SCC*mec*	SH621-AB478934.1	90.39	333	28	3	8212	8542	3291	2961	4.74E-122	435
SH51-SCC*mec*	SH621-AB478934.1	89.458	332	29	5	8334	8662	3449	3121	6.18E-116	414
SH51-SCC*mec*	SH621-AB478934.1	89.003	291	32	0	8372	8662	3451	3161	8.16E-100	361

SH51-SCC*mec*	SH621-AB478934.1	89.328	253	23	3	8212	8462	3211	2961	6.45E-86	315
SH51-SCC*mec*	SH621-AB478934.1	88.095	252	26	3	8413	8662	3450	3201	2.34E-80	296
SH51-SCC*mec*	SH621-AB478934.1	88.732	213	22	2	8214	8425	3169	2958	3.06E-69	259
SH51-SCC*mec*	SH621-AB478934.1	88.304	171	16	3	8494	8662	3449	3281	5.24E-52	202
SH51-SCC*mec*	SH621-AB478934.1	87.861	173	17	3	8212	8382	3131	2961	1.88E-51	200
SH51-SCC*mec*	SHATCC29970-NZ_CP035291.1:1895881-192102	99.897	1938	1	1	14087	16023	17362	15425	0	3567
SH51-SCC*mec*	SHATCC29970-NZ_CP035291.1:1895881-192102	99.574	1876	5	3	14080	15953	12129	14003	0	3417
SH51-SCC*mec*	SHATCC29970-NZ_CP035291.1:1895881-192102	100	78	0	0	15946	16023	17401	17478	8.95E-35	

Table S1-2 Comparative analyses of phage-related fusidic acid resistance islands betweenSH51 and other reference genomes using the blastn mode of the BLAST+ command-line suite.

query id	subject id	% identity	alignment length	mismatches	gap openings	q.start	q.end	s.start	s.end	e-value	bit score
SH51-*fusB*	SeRIfusB-2793-JF777505.1	98.58	1972	26	1	15011	16980	14999	16970	0	3485
SH51-*fusB*	SeRIfusB-2793-JF777505.1	91.642	335	24	4	4314	4646	4160	4492	3.98E-130	460
SH51-*fusB*	SeRIfusB-704-JF808725.1	99.711	2073	1	3	15557	17624	14538	16610	0	3790
SH51-*fusB*	SeRIfusB-704-JF808725.1	87.475	495	55	5	4314	4806	3484	3973	2.95E-161	564
SH51-*fusB*	SeRIfusB-704-JF808725.1	88.477	243	26	1	15011	15251	14321	14563	1.54E-79	292
SH51-*fusB*	SeRIfusB-5907-JF777506.1	99.752	1211	3	0	15770	16980	15560	16770	0	2220
SH51-*fusB*	SeRIfusB-5907-JF777506.1	91.642	335	24	4	4314	4646	4208	4540	3.98E-130	460
SH51-*fusB*	SeRIfusB-5907-JF777506.1	88.272	162	19	0	15011	15172	15409	15570	4.46E-50	195
SH51-*fusB*	NTUH-3692-AB828059.1	99.17	1808	8	5	15824	17624	13214	15021	0	3249
SH51-*fusB*	NTUH-3692-AB828059.1	80	1590	273	32	676	2232	237	1814	0	1133
