# Supplementary material for: Multidrug-Resistant Staphylococcus haemolyticus ST42 Carrying ΨSCCmec57395-like SCCmec and Resistant Islands with Type I aj1–LP–fusB Structure Emerges in Taiwan Hospitals
Source: Antibiotics (Basel). 2025 Oct 13;14(10):1015. doi: 10.3390/antibiotics14101015 (PMC12561750; doi:10.3390/antibiotics14101015)
Supplement: Supplementary file 1 [file antibiotics-14-01015-s001.zip › Table S2.pdf]

Table S2. Primers used in this study.

Primer set	Primer name	Sequence (5′ to 3′)	Expected size (bp)	References
**SCC*mec* Cassette**				
A1(ST3)	orfX-F1	GAAAAAGCACCTGAAAATATGAG	8568	This study
	hel-R1	GATAAAACCTTTATGGGCTCTATC		
A2(ST3)	hel-F1	CACGTGATAGAATACATCGTTTAG	4339	This study
	AALP-R	TGCGTTAGTCCAAGTCAATC		
A3(ST3)	AALP-F	TTAATTGGCTGGGTAGGTCA	2179	This study
	IS431 P4	CAGGTCTCTTCAGATCTACG		
A4(ST3)	SCCmec-M2-IS2IS2	TGAGGTTATTCAGATATTTCGATGT	5848	This study
	cadD-R1	CTACCGCAGTTGCAATATAAAG		
A5(ST3)	cadD-F1	GTTGAGAAGTTCAGTCGTTGG	3102	This study
	SCCmec-M2-IS2IS2	TGAGGTTATTCAGATATTTCGATGT		
A6(ST42)	orfX-F1	GAAAAAGCACCTGAAAATATGAG	647	This study
	cadD-R1	CTACCGCAGTTGCAATATAAAG		
B(ST3/42)	IS431 P4	CAGGTCTCTTCAGATCTACG	1851	This study

	MecA147-R	ATGCGCTATAGATTGAAAGGA		
C(ST3/42)	MecA147-F	GTGAAGATATACCAAGTGATT	3693/3678	This study
	SCCmec-M2-IS2IS2	TGAGGTTATTCAGATATTTCGATGT		
D(ST3/42)	IS431 P4	CAGGTCTCTTCAGATCTACG	3069/3164	This study
	attSCC-R	ATATGCTTCTGCGTATCG		
fusB detection				
	fusB531-559F	CGGATGGTCAATATGTAAAAAAAGGTGAC		[26,29]
	fusBR	ACAATGAATGCTATCTCGACA		
fusC detection				
	fusC162-183F	GGACTTTATTACATCGATTGAC		[26,29]
	fusC572-550R	CTGTCATAACAAATGTAATCTCC		
**Phage related resistant island**				
smpB-int detection	ssra407-429 (F)	TCAAGCACTTAAAGAAAAAGCGG	1483	[28,29]
	Int(III) 175-194F	GACGAGTTAGAGGGTATTGG		
groEL-int detection	groEL 1213-1232F	GTKGAAGAAGGTATYGTTGC		[27]

	int(I) 109-128F	CGTAAATCAGACGCTAAACA		
rpsR-*int* detection	rpsR 6-24F	AGGTGGACCAAGAAGAGGC		[27]
	int(II) 541-565F	GCTAAACGTAATAACTATTTAGAAG		
aj1-LP-*fusB* detection	aj1606-557R	AGTAAAGAATAAGTTTTTAATCGTTAATGC	1467	[27]
	fusB 389-361R	TTCCGATTTGATGCAAGTTCATTCCATCC		
